# The Utility of Iron Chelators in the Management of Inflammatory Disorders

**DOI:** 10.1155/2015/516740

**Published:** 2015-03-23

**Authors:** C. Lehmann, S. Islam, S. Jarosch, J. Zhou, D. Hoskin, A. Greenshields, N. Al-Banna, N. Sharawy, A. Sczcesniak, M. Kelly, K. Wafa, W. Cheliak, B. Holbein

**Affiliations:** ^1^Department of Anesthesia, Pain Management and Perioperative Medicine, Dalhousie University, 5850 College St., Halifax, NS, Canada B3H 1X5; ^2^Department of Microbiology and Immunology, Dalhousie University, 5850 College Street, Halifax, NS, Canada B3H 1X5; ^3^Department of Pharmacology, Dalhousie University, 5850 College St., Halifax, NS, Canada B3H 1X5; ^4^Department of Pharmacy, East West University, Plot No. A/2, Jahurul Islam City, Aftabnagar, Dhaka 1219, Bangladesh; ^5^Department of Pathology, Dalhousie University, 5850 College St., Halifax, NS, Canada B3H 1X5; ^6^Department of Physiology, Faculty of Medicine, Cairo University, 1 Al-Saray St., Cairo 11559, Egypt; ^7^Chelation Partners Inc., 1411 Oxford St., Halifax, NS, Canada B3H 3Z1

## Abstract

Since iron can contribute to detrimental radical generating processes through the Fenton and Haber-Weiss reactions, it seems to be a reasonable approach to modulate iron-related pathways in inflammation. In the human organism a counterregulatory reduction in iron availability is observed during inflammatory diseases. Under pathological conditions with reduced or increased baseline iron levels different consequences regarding protection or susceptibility to inflammation have to be considered. Given the role of iron in development of inflammatory diseases, pharmaceutical agents targeting this pathway promise to improve the clinical outcome. The objective of this review is to highlight the mechanisms of iron regulation and iron chelation, and to demonstrate the potential impact of this strategy in the management of several acute and chronic inflammatory diseases, including cancer.

## 1. Introduction

Although iron exists in abundance in the earth's crust and the environment, iron is found in relatively low concentrations in aqueous systems under aerobic conditions. Fe occurs in two main oxidation states: the reduced Fe^2+^ (Fe II, ferrous) and the oxidized Fe^3+^ (Fe III, ferric) form. Iron represents an essential trace element for almost all forms of life; however, iron has paradoxical properties. It readily accepts and donates electrons, converting between the more soluble ferrous form and the insoluble ferric form, and thus plays an integral role in electron transfer and oxygen transport as well as adenosine triphosphate and deoxyribonucleic acid synthesis [1]. However, iron can also catalyze the formation of reactive oxygen species (ROS) via redox reactions. The Fenton and Haber-Weiss reactions of H_2_O_2_ with Fe^2+^ generate hydroxyl radicals that promote oxidative stress and are responsible for lipid, protein, and DNA damage. Importantly, dysregulated iron homeostasis is associated with progressive inflammatory and degenerative diseases, as well as cancer [2]. Iron and its homeostasis are intimately tied to the inflammatory response. Iron withdrawal is part of the natural innate immune response in inflammation [3]. During inflammation and infection a “hypoferremic response” is observed (anemia of inflammation) [4].

Given the role of iron in development of inflammatory diseases, pharmaceutical agents targeting this pathway promise to improve the clinical outcome. The objective of this review is to highlight the mechanisms of iron regulation and iron chelation and to demonstrate the potential impact of this approach in the management of several acute and chronic inflammatory diseases, including cancer. For this purpose, we reviewed the literature regarding experimental and clinical evidence for iron-related anti-inflammatory strategies and discuss implications and limitations of iron removal in inflammation.

## 2. Basic Mechanisms

### 2.1. Iron Absorption and Regulation in the Human Body

Dietary iron uptake is closely regulated, which is critical to cell physiology and to ensure minimal concentrations of potentially dangerous free intracellular iron. Mechanisms for iron homeostasis are complex compared to other metals, which are typically controlled by a simple elimination process [5]. Iron requirements are high during infancy, childhood, and pregnancy [6]. Absorption declines to around 1 mg/day in men and 2 mg/day in women when growth declines [5]. Both heme and nonheme iron can be utilized by the intestinal epithelium. Heme iron is abundant in meat as hemeprotein and myoglobin, released from hemeprotein by proteolytic enzymes in the stomach and small intestine. Nonheme iron crosses the apical brush border membrane of enterocytes after conversion into ferrous iron by duodenal cytochrome B [7]. Iron has two fates according to the requirement of the body once within the enterocytes. When iron demand in the body is low, iron remains sequestered in the enterocyte within ferritin as a mechanism of iron storage. When the iron demand of the body is high, iron crosses the basolateral membrane via the iron export protein ferroportin1 (FPN) and enters the circulation, ultimately binding to transferrin. FPN is found in the basolateral membrane of the enterocytes and in large quantities on macrophages [8]. Ferroxidase activity of ceruloplasmin or hephaestin is required to load iron safely onto transferrin [9]. To mitigate iron losses from the body from shedding of epithelial cells and menstruation, the body must absorb an equivalent amount of iron from the gut, maintaining an overall iron balance.

In total, the human body contains approximately 3 to 4 g of iron in the form of both heme and nonheme iron [10]. Human hemoglobin accounts for 65% of total body iron, while 25% of body iron is bound to iron storage proteins ferritin and hemosiderin. The remaining 10% are constituents of myoglobin, cytochrome, and other iron-containing enzymes [11]. Only about 0.1% of body iron is bound to transferrin and this circulates as a soluble exchangeable pool in the plasma. Tissue macrophages phagocytize senescent red blood cells to recover, turnover, and recycle iron. This recycling of iron by macrophages is 20-fold greater than the amount of iron absorbed daily by the intestine. Iron absorption by enterocytes and the recycled iron efflux from macrophages are controlled by hepcidin, a key iron regulatory hormone produced by hepatocytes [12, 13]. It limits the cellular iron efflux by binding to FPN and facilitates its internalization and ultimate degradation [14]. Hepcidin expression is increased in times when iron stores are sufficient and is decreased when the requirement of iron is high. Poor gut iron absorption can be predicted by elevated hepcidin level [15]. The regulation of iron absorption is critical because humans do not have physiological pathways for its excretion. Hepcidin levels are increased during infection and inflammation. Its release is upregulated in response to inflammatory cytokines, in particular interleukin-6 (IL-6) [16–18]. Hepcidin is also produced by macrophages and neutrophils during the innate immune response through a Toll-like receptor 4-dependent pathway [19]. In contrast, the level of hepcidin decreases during hypoxia, during iron deficiency, and when erythropoietic needs are increased [5].

### 2.2. Iron and ROS

**Figure 1:**
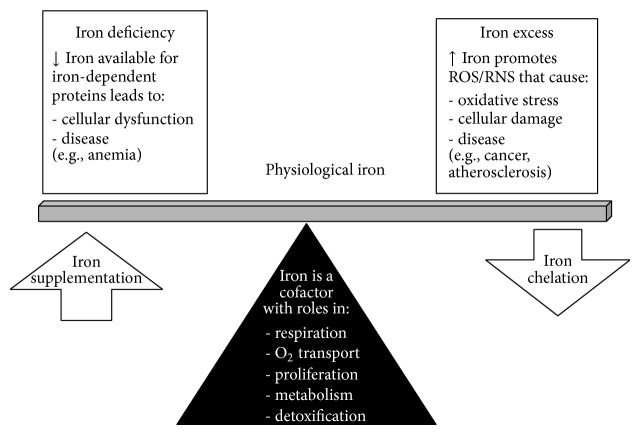
Role of iron in the development of disease.

**Figure 2:**
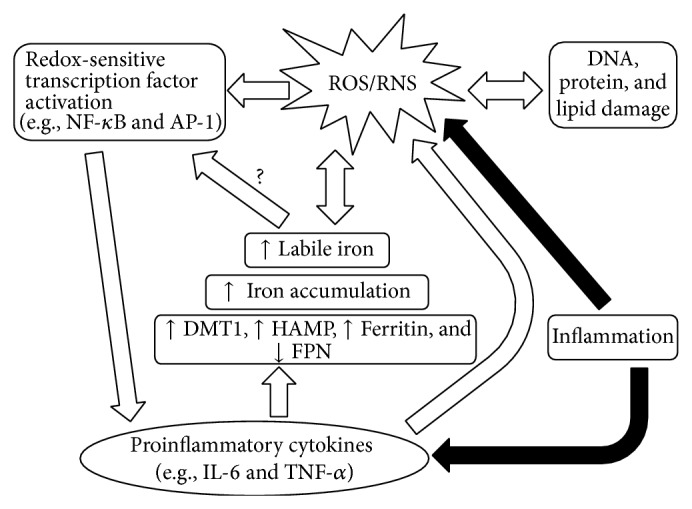
Putative involvement of iron in inflammation. Inflammation induces the production of proinflammatory cytokines which can induce the sequestration of iron within cells through the modulation of iron regulatory protein expression (e.g., divalent metal transporter 1 (DMT1), hepcidin (HAMP), ferritin, and ferroportin1 (FPN)). Increased iron levels, especially in macrophages, may lead to ROS production via the Fenton and Haber-Weiss reactions. ROS in return can increase levels of labile iron and induce oxidative damage of DNA, proteins, and lipids. Additionally, ROS can activate redox-sensitive transcription factors whose targets include proinflammatory cytokines.

ROS possess paradoxical cellular effects, as they can both induce cell growth and survival or trigger cell death. Multiple factors influence the cellular effects of ROS, including ROS type as well as the level, localization, and duration of the assault [20–22]. Low levels of ROS act as second messengers in many important signaling pathways, while prolonged exposure to higher levels can exceed cellular antioxidant defenses and damage DNA, lipids, and proteins [21, 23]. A complex relationship exists between iron metabolism and oxidant status (Figures 1 and 2). Free iron plays an important role in ROS production as it can participate in the Fenton and Haber-Weiss reactions to yield superoxide anion and the highly reactive hydroxyl radical [20–22]. To prevent iron-catalyzed ROS production, iron levels are strictly regulated by multiple factors including hepcidin and FPN, as described above.

During inflammation, various pathways in immune cells are involved in the generation of ROS and RNS [24]. ROS and RNS generation can lead to significant tissue damage and is responsible for a variety of inflammatory disease processes [20–22]. Proinflammatory cytokines induced by inflammation can stimulate anemia of inflammation by limiting serum iron and increasing cellular iron stores by modulating the expression and activity of various iron regulatory proteins including hepcidin, FPN, ferritin, and the iron importer divalent metal transporter 1 (DMT1) [25–30]. Due to their role in iron recycling from erythrocytes, iron loading in macrophages is particularly significant [31]. Iron accumulation, in addition to increased levels of ROS and RNS induced by other facets of inflammation, may lead to increased labile iron, which can further perpetuate ROS production and damage through participation in the Fenton and Haber-Weiss reactions (Figure 2). In addition, increased labile iron can contribute to the activation of redox-sensitive transcription factors such as NF-*κ*B, which is a potent regulator of inflammatory gene products including TNF-*α* [32]. An inhibitory effect on NF-*κ*B-mediated generation of TNF-*α* and other inflammatory cytokines has been reported in Kupffer cells following the reduction of free iron by chelation [33].

## 3. Iron Chelators

### 3.1. Chemistry of Iron and Iron Chelators

ROS and RNS are produced both during normal aerobic respiration of cells and at increased rates and concentrations as part of the inflammatory response. Of the various reactive species, ROS have free iron directly involved in their production through the Fenton and Haber-Weiss reactions. Thus, the harnessed and controlled redox cycling of iron, which contributes activity for cell-essential iron-dependent enzymes such as the cytochromes, myeloperoxidase, or superoxide dismutase, also provides a less controlled source of cell-damaging reactive chemicals; that is, should free iron be available for Fenton or Haber-Weiss chemistry, the latter comprises essentially side reactions.

Iron can achieve hexadentate coordination with electron-donating ligands and when fully coordinated it is relatively redox stable. Studies have shown that Fenton-induced hydroxyl radical formation requires at least one free coordination site on the catalyzing iron [34]. Moreover, these studies were done using various chelating molecules with varying abilities to coordinate up to the six available coordination sites on iron and these therefore illustrated a key feature of iron chelators. For example, iron-EDTA and iron-bleomycin chelates are redox-active liberating radicals while the hexadentate Fe-desferal chelate is relatively redox-stable [34]. By definition (IUPAC, 1997) a metal chelate includes at least two coordinate bonds with a metal contributed by a chelating ligand and this, therefore, has important implications for the selection and use of iron chelators for treating inflammation or other disease conditions. It is interesting to note that the transferrins which are the main vertebrate iron chelator and transport protein molecules hold iron protected, within the transferrin structure itself, fully coordinating bound iron in a stable form using carbonate as a coligand [35]. Iron has a hexadentate coordination capacity. Noncoordinated sites have a high reactivity. Therefore, inappropriate or weak chelating molecules that retain their chelated iron with free, otherwise redox reactive coordination sites could be problematic as to undesirable radical generation. This issue might be further compounded on the basis that such chelators might solubilize and hold additional reactive iron spatially available at or near sensitive sites or, alternately, might deliver reactive iron to sensitive sites for participation in hydroxyl radical formation. An example of this problem is evident with the aminoglycoside antibiotics used to treat bacterial infection but known to possess ototoxicity related to oxidative radical formation when these agents bind iron in a redox active state [36].

### 3.2. Anti-Inflammatory Chelators, Designer Molecules, and Other Pharmaceutical Agents as Iron Chelating Anti-Inflammatory Agents

#### 3.2.1. Natural Products

Desferal (Novartis), also known as desferrioxamine or deferoxamine B, is a natural microbial product and an excellent example of a siderophore produced by microbes, in this case by* Streptomyces* spp. [37]. Both pathogenic bacteria [38] and fungi [39] can produce iron chelating siderophores as one strategy for obtaining host iron that is essential to their growth and pathogenesis [37, 39]. Iron withholding and microbial acquisition are key aspects of the host parasite battle during the pathogenesis of infection [40, 41]. While desferrioxamine reduced experimental LPS-induced inflammation in mice, its use and that of other microbial chelators need to be carefully considered given that these agents could potentially promote infection owing to their microbial origin, for example, as has been shown for desferrioxamine with* Yersinia enterocolitica* [42] or* Candida albicans* [43].

A variety of natural phytochemicals have been described as having anti-inflammatory activity and some of these have been shown to possess iron-chelating activity [44]. For example, curcuminoids bind ferric iron* in vitro* and alleviate iron toxicity in thalassemic mice [45, 46]. In addition, Aayush et al. [47] recently reviewed the iron chelating activity of African walnut and wheat grass extracts as to their potential for natural iron removal agents for iron overload associated with thalassemia, which is currently typically treated clinically with desferal. However, the role that iron chelation plays with various phytochemicals in relation to their anti-inflammatory activities remains unclear.

#### 3.2.2. Synthetic Compounds

As to synthetic agents with anti-inflammatory activity, ibuprofen, a widely used synthetic anti-inflammatory agent, has been shown to chelate iron in a stable form without a free Fenton-reactive coordination site and to protect from lipid peroxidation* in vitro* and phosgene-induced septic lung injury in rabbits [48]. This evidence suggests at least part of ibuprofen's mode of action is related to iron chelation and suppression of ROS activity.

Research on newer generation synthetic iron chelators has produced a number of candidate molecules primarily in relation for their potential for treating iron overload or as anticancer agents. In this regard, agents that hold iron in a ROS-reactive manner have been reviewed, their enhanced ROS being proposed to provide killing of cancer cells [49]. Such ROS-reactive molecules would not likely provide anti-inflammatory activity but, conversely, could induce inflammatory responses.

On the other hand, new iron chelators that bind iron in a stable manner would be useful for treating iron overload diseases and thus, presumably, inflammation. Kalinowski and Richardson have recently reviewed synthetic chelators based on various chelating chemical groupings such as catechol, hydroxamate, and hydroxypyridinone, concluding that structures providing hexadentate hydroxypyridinone functionality (an example being deferiprone) have particular promise as bacteriostatic agents in relation to outcompeting siderophores of microbes [49]. However, the hydroxamate desferal and the hydroxypyridinone deferiprone have been shown to be accessible by various micoorganisms thereby restricting their potential use as microbial control agents [50]. Holbein and Mira reported that DIBI, a new chelator, which possesses modified hydroxypyridinone activity, provided Fe-specific growth inhibition of* Candida albicans* [43]. DIBI is one example of a new approach to providing controlled molecular weight chelating-functional polymers. These agents may provide an additional advantage through providing compartmentalized sinks for iron thus reducing its participation in ROS inflammatory reactions or its bioavailability to either pathogenic cancer or microbial cells.

## 4. Experimental Studies

### 4.1. Cancer

Deficient regulation of iron homeostasis can contribute to tumor development through a number of different mechanisms (Figure 1, Table 1). The iron-catalyzed production of ROS and subsequent damage to DNA can result in the loss of tumor suppressors and activation of oncogenes [51, 52]. Oxidative stress may also modulate signal transduction pathways associated with malignancy [53]. Cancer cells require considerably more iron than normal cells due to their increased rate of DNA synthesis and utilization of the iron-dependent enzyme ribonucleotide reductase [54]. Thus, cancer cells have increased expression of transferrin receptor-1 and a higher rate of iron uptake from transferrin [55, 56], as well as decreased expression of iron exporters [57]. In addition, iron regulates the activity of the transcription factors NF-*κ*B and HIF-1*α* [58], which promote the expression of genes involved in the survival and metastasis of cancer cells [59, 60]. Interestingly, higher concentrations of free iron in breast cancer cells are associated with a more aggressive tumor phenotype [57]. It follows that cancer cells are more sensitive to iron deprivation than normal cells and may therefore be susceptible to treatment with iron chelators [61].

### 4.2. Atherosclerosis

Atherosclerosis is characterized by chronic vascular inflammation [74]. It has been suggested that the development of atherosclerosis is associated with the amount of iron stored in the body and iron may contribute to the pathogenesis of atherosclerosis by acting as a regulator of vascular oxidative stress and inflammatory immune responses in atherosclerosis [75]. Increased levels of iron and oxidized lipids are both found in high-cholesterol diet-fed rabbits associated with atherosclerotic lesions [62]. Iron chelation by deferoxamine reduced the expression of oxidative stress markers and delayed the formation of atherosclerotic lesions, indicating that chelation therapy may aid in prevention of atherosclerosis [63, 76].

### 4.3. Diabetes and Obesity

Diabetes mellitus is characterized by an impaired glucose metabolism with the main symptom of hyperglycemia, caused either by impaired insulin secretion or impaired insulin action or both [77]. Obesity is characterized by an increase in the number and/or the size of the fat cells [78]. By activating adipocytes and stimulating their growth, iron contributes to obesity. Iron reduction by deferoxamine resulted in amelioration of adiposity via the regulation of oxidative stress and inflammation in obese and type 2 diabetes mice [65]. Interestingly, despite of deferoxamine administration for two weeks, there was only a mild but not significant reduction in the haemoglobin concentration and the hematocrit between vehicle-treated and chelator-treated mice (Table 1).

The uptake of unbound, that is, non-transferrin-bound iron, into pancreatic beta cells causes oxidative stress via the Fenton reaction. Due to their reduced antioxidant capacity, cell death is induced in the beta cells of isolated pancreatic islets by pharmacological-relevant iron concentrations that may occur during intravenous iron supplementation [66].

### 4.4. Renal Fibrosis

Renal interstitial fibrosis is characterized by the accumulation of collagen and related molecules in the interstitium, involving cells like tubular epithelial cells, fibroblasts, fibrocytes, myofibroblasts, monocyte/macrophages, and mast cells [79], and can be caused inter alia by inflammatory processes [80]. In a recent study, the impact of deferoxamine on experimentally induced renal fibrosis was explored, again with an emphasis on inflammation caused by iron induced oxidative stress. After a surgically induced unilateral ureter obstruction (UUO), mice treated with DFO showed significantly less fibrotic progression, less interstitial macrophage infiltration, and thereby a reduced expression of IL-1*β* and MCP-1 and a suppressed UUO-induced accumulation of myofibroblasts, compared with untreated mice. The authors concluded that in mice iron reduction by deferoxamine may prevent renal interstitial fibrosis by regulating TGF-beta/Smad signaling, oxidative stress, and inflammatory responses [67].

### 4.5. Glaucoma

Elevated intraocular pressure, the main risk factor for glaucoma, triggers the initiation and progression of oxidative stress-induced cell damage [81]. The disease is marked by loss of optic nerve axons and retinal ganglion cells, resulting in characteristic optic nerve atrophy and visual field defects [69]. Wang et al. observed a correlation of iron intake and the odds of glaucoma in humans [68].

In an* in vivo* model in rats, chelation treatment ameliorated ocular sequelae caused by increased intraocular pressure. The topically administered metal chelator, EDTA, combined with a permeability enhancer reduced signs of oxidative stress and inflammation in glaucoma in the rat's eyes, increased retinal ganglion cell survival, and decreased demyelination of optic nerve compared with untreated eyes [69].

### 4.6. Systemic Inflammatory Response Syndrome

Systemic inflammatory response syndrome (SIRS) is characterized by specific physiological alterations, including temperature, white blood cell count, heart rate, and respiratory rate, caused by a broad spectrum of noninfectious and infectious triggers [82]. Hypoferremia in SIRS is observed in humans [83] and animals [84, 85]. In acute hepatic ischemia induced SIRS and consecutive multiple organ dysfunction (MOD) in pigs, Vlahakos et al. [70] demonstrated that DFO attenuated lipid peroxidation, inhibited IL-6 production, and substantially diminished SIRS and MOD. Tubulointerstitial damage in the porcine kidney as expression of severity of SIRS induced organ failure was reduced by a bolus followed by a continuous infusion of the chelator. These results suggest that iron plays a pivotal role in the pathogenesis of SIRS and MOD including acute kidney injury by its involvement in various inflammatory pathways and in the generation of reactive oxygen species. Using the same model, the researchers also demonstrated that application of DFO significantly reduced brain edema, intracranial pressure, and lung injury [71, 72].

In an endotoxemic mouse model, lactoferrin, a nonheme iron-binding glycoprotein, decreased LPS-induced oxidative burst and reactive oxygen species in cultured cells and attenuated mitochondrial dysfunction in liver of endotoxemic mice [86]. In a carbon tetrachloride induced acute hepatic injury rat model, application of DFO significantly decreased oxidative stress and limited inflammatory infiltration and hepatocyte necrosis, resulting in reduced mortality rate [87].

### 4.7. Ischemia Reperfusion Injury

Ischemia reperfusion injury can be regarded as the most exaggerated form of oxidative stress for cells [88]. ONOO^−^ and H_2_O_2_ produced in the reperfusion phase are known to release iron ions from intracellular iron-sulphur proteins thus increasing intracellular labile iron pool, which in turn promotes by the above-outlined reactions the production of further reactive species [89].

In the postischemic renal injury model in rats, acute iron loading exacerbated postischemic lipid peroxidation and renal injury, while reducing iron level by DFO suppressed lipid peroxidation and improved renal function [90]. In a cardioplegia-ischemia and reperfusion model via cardiopulmonary bypass in sheep, DFO protected lung injury by inhibiting endothelial injury and eliminating postischemic cardiac stunning [91].

### 4.8. Colitis

Colitis is another example for chronic inflammation, leading to anemia of inflammation as a consequence of low serum iron and low iron-binding capacity [92].

Using a trinitrobenzene sulfonic acid induced colitis rat model, Minaiyan and coworkers compared the effect of deferiprone and deferoxamine with the newer iron chelators, maltol and kojic acid, on inflammatory response. In the highest dosage maltol was comparable with prednisolone as standard anti-inflammatory drug and also deferoxamine and deferiprone as reference iron binding agents [73].

## 5. Clinical Evidence

### 5.1. Iron Homoeostasis in Inflammation

#### 5.1.1. Anemia of Inflammation

Anemia represents a common clinical finding in patients with acute or chronic inflammation. In acute inflammation (e.g., trauma and surgery) the two major mechanisms leading to anemia are blood loss and blunted erythropoiesis due to decreased iron availability [93, 94]. In the event of chronic blood loss and/or persisting decreased iron absorption, acute anemia may evolve to anemia of chronic disease (ACD) with true iron deficiency (ACD+ID).

Anemia in chronic inflammation (without blood loss or decreased baseline iron availability) represents a specific entity, that is, ACD. It is observed, for example, in cancer, rheumatoid arthritis, inflammatory bowel diseases, and congestive heart failure, as well as in sepsis and chronic renal failure. This anemia is the result of activation of the immune system by the underlying process and certain immune and inflammatory cytokines including tumor necrosis factor-alpha, interferon-gamma, IL-1, IL-6, IL-8, and IL-10 [4, 95].

#### 5.1.2. Hepcidin

The discovery of hepcidin yielded significant insight into the link between the immune response in inflammation and systemic iron homoeostasis. The above-mentioned cytokines are known to increase hepcidin expression. Circulating hepcidin expression* in vivo* has also been correlated with acute phase proteins such as C-reactive protein, *α*-1-acid-glycoprotein, ferritin, and amyloid A [96, 97].

Hepcidin produced by the hepatocytes binds to its receptor FPN, which is an iron export protein that is present on limited cells such as macrophages, hepatocytes, duodenal enterocytes, and placental syncytiotrophoblasts [98]. Hepcidin binding to ferroportin leads to ubiquitinization, followed by internalization and degradation of FPN [98]. The end result is that iron cannot be released into the plasma and remains trapped inside the macrophages and hepatocytes, resulting in an increase in iron stores reflected in high levels of serum ferritin [29]. In addition, hepcidin inhibits intestinal absorption of iron [99]. Therefore, ACD is characterized by low serum iron, transferrin, and total iron binding capacity, by normal transferrin saturation, and by increased ferritin, the latter in contrast to iron deficiency anemia. The low transferrin levels are due to downregulation of transferrin synthesis as a result of an increase in ferritin.

The increase in hepcidin production in response to inflammation is a protective mechanism in the case of infections in which iron restriction would limit bacterial growth. However, in ACD or inflammation without infection, this mechanism can have detrimental consequences when iron remains sequestered in the macrophages and hepatocytes and is not available for erythropoiesis, resulting in anemia [97].

### 5.2. Preexisting Diseases with Iron Overload

#### 5.2.1. Hereditary Iron Storage Diseases

Iron overload can be the result of hereditary (primary) or acquired (secondary) increase in iron storage. The most common hereditary iron storage disease is hemochromatosis. Most forms of hemochromatosis result from dysregulation of hepcidin or defects of hepcidin or ferroportin themselves [100]. When hepcidin binds to ferroportin it causes internalization of ferroportin and its proteolytic destruction [17]. Thus, hepcidin serves to prevent the egress of iron both from intestinal cells and from macrophages. Hepcidin is normally upregulated by excess iron stores [17]. This serves to prevent further absorption of iron from the gastrointestinal tract and its release from macrophages. It is this regulation that is impaired in several types of hemochromatosis [100]. There is evidence that patients with hemochromatosis have a higher risk for infectious diseases. Iron overload associated with hereditary hemochromatosis has been reported to confer susceptibility to infectious pathogens, such as* Yersinia enterocolitica* and* Vibrio vulnificus* [101].

#### 5.2.2. Acquired Iron Storage Disease

Acquired iron overload is frequently observed in thalassemia, myelodysplastic syndromes, congenital dyserythropoietic anemias, sickle cell disease, and other hemoglobinopathies. Patients with thalassemia, whose erythroid precursor populations are greatly expanded but fail to mature into functional erythrocytes, have increased intestinal iron absorption despite often severe systemic iron overload [102]. Although blood transfusions given for severe anemia (e.g., thalassemia major) contribute to the lethal iron overload in ineffective erythropoiesis, many patients with less severe anemia (e.g., thalassemia intermedia) receive few or no transfusions but still become severely iron-overloaded [103]. Walter et al. found increased levels of plasma malondialdehyde in thalassemia [104]. They described three potential mechanisms: (i) the excess *α*-chains in *β*-thalassemic erythrocytes and erythroblasts being unstable and prone to denaturation and oxidation, (ii) peroxidation of tissues that leak malondialdehyde into the blood, and (iii) depleted antioxidant capacity lowering defense against oxidants [105]. Infections and inflammations are more frequent in thalassemic patients with iron overload induced by frequent blood transfusions [104].

#### 5.2.3. Other Diseases with Iron Involvement

Iron accumulation and increased oxidative stress were also described in the pathogenesis of preeclampsia [2], diabetes [106], the metabolic syndrome [107], obesity [108], hypertension [109], cardiovascular diseases [110], heart failure [95], atherosclerosis [111], stroke [112], Alzheimer's, Parkinson's and other major neurodegenerative diseases [113], Friedreich's ataxia [114], amyotrophic lateral sclerosis [115], rheumatoid arthritis [116], systemic lupus erythematosus [117], asthma [118], inflammatory bowel diseases [119], age-related macula degeneration [120], psoriasis [121], gout [122], chronic obstructive pulmonary disorder [123], cancer [51], malaria [124], and other diseases (for overviews see Kell 2009 [2] and Weinberg 2010 [125]).

### 5.3. Preexisting Diseases with Iron Deficiency

Iron deficiency (ID) can be caused by inadequate oral iron uptake (dietary), inadequate iron absorption (e.g., celiac disease), excessive blood loss, or increased iron demand (e.g. growth or pregnancy). The clinical picture of ID patients is usually characterized by mild to severe anemias and further consequences, including epithelial changes (stomatitis and glossitis), and neurocognitive effects, that is, impaired motor and mental functioning [126]. Imperative to treatment is identifying the underlying cause of iron deficiency. Oral iron replacement is preferentially used to replete iron stores. Ferrous sulfate is the most common oral iron supplement used [126].

However, in the same way that primary or secondary iron storage diseases can cause oxidative stress, artificial iron overload due to therapeutic administration can cause harm for the patients. The Pemba trial, which led to adverse events in children receiving iron in a malaria-endemic region, underscores the need for caution [127]. For tuberculosis, it has been demonstrated that parenteral or oral iron increase mycobacterial growth [128] and that morbidity and mortality increase in patients receiving iron supplementation [129]. In fact, dietary iron is associated with occurrence and death from tuberculosis [130].

Mild ID has been associated with protection against certain inflammatory and infectious conditions. The “iron hypothesis” of the benefits of some iron depletion due to menstruation was devised to account for the lowering of heart-disease risk in young women that disappears in those who are postmenopausal [131, 132].

### 5.4. Clinical Iron Removal in Inflammation

#### 5.4.1. Potential Indications

Depending on the localization and severity of the inflammatory condition, different approaches and routes of administration for iron chelators are feasible. The route of administration as well as needs for extra- and/or intracellular action determines the required molecular structure and weight of the iron chelating substances. For wound infections, topical/local administration of large molecular size substances might be feasible, whereas for systemic (SC/IV/IP) administration (e.g., sepsis) smaller molecules might be necessary. Due to the bacteriostatic/bactericidal effects of iron removal from media, the use of iron chelators as preservatives in standard medications (e.g., eye drops) is also an option. In the same way, application of iron chelators as adjunct to peritoneal lavage fluids appears to be an option for both prophylactic and therapeutic purposes.

#### 5.4.2. Potential Limitations

Suggesting that inflammatory disorders might be treatable by the induction of a second disorder, that is, iron depletion, might be seen as a controversial proposal due to the prevalent assumption that iron in storage is inherently safe. This assumption is based on traditional medical practices rather than on rigorous clinical trials [133]. It has been previously stated that the benefit of iron depletion can only be rigorously demonstrated in relation to the state of iron in excess of needs (i.e., the condition of having iron in storage). It has not been widely appreciated that the safety of stored iron can only be shown with clarity in studies of the same design. Absence of proof that iron depletion is beneficial implies an absence of proof of the safety of stored iron. Because of the deeply rooted assumption that stored iron is safe, appropriate trials to rule out the potential hazards of iron stores have not been undertaken [134]. But even if there exists a potential (short-term) harm of iron depletion (e.g., anemia) when administered in inflammatory diseases, this can be compared with “side effects” of chemotherapy in cancer: “collateral damage” appears acceptable, if it is possible to get control over the main, potentially life-threatening condition.

## 6. Conclusions

Iron is involved in almost every clinical condition of acute or chronic inflammation. Since iron can contribute to detrimental ROS and RNS generating processes, it seems to be a reasonable approach to modulate iron-related pathways in inflammation. In humans (with normal baseline iron levels), a counterregulatory reduction in iron availability is observed during inflammatory diseases (anemia of inflammation). Under pathological conditions with reduced or increased baseline iron levels different consequences regarding protection or susceptibility to inflammation have to be considered.

Therapeutic interventions with iron or iron chelators have an impact on the oxidant status and iron chelation might be utilized as a potential antioxidative strategy in inflammatory diseases. New iron chelators designed to sequester iron in a nonredox active state may prove to provide increased efficacy in this regard. Additional requirements for new iron chelators apply in infection-induced, local, and systemic inflammation. Conventional clinical chelators do not effectively deny iron to pathogens; in fact pathogens can utilize these conventional chelators as iron sources. This underscores the need for superior chelators since agents like desferal or deferiprone as antimicrobials agents have provided varying results depending on the microbe being tested.

## Figures and Tables

**Table 1:** Selected experimental studies.

Disease	Subject	Results	Reference
Cancer	Analysis of the specific murine OKT9-antibody on human leukemia cells	OKT9 receptor is transferrin OKT9 binding on both normal and malignant cells was strongly associated with proliferation	[55]
	Expression of ferroportin and hepcidin in cultured human breast cancer cells plus an observational cohort study in patients	Reduction of ferroportin in cancer cells altered the labile iron pool Combined ferroportin/hepcidin gene expression identified clinical subset of breast cancer	[58]

Atherosclerosis	Influence of zinc to the development of atherosclerotic plaques	Zinc fed rabbits showed decreased atherosclerotic plaques Average lesion Fe levels in the zinc-fed group were significantly higher	[62]
	Effect of iron chelation on ferritin induction and iron accumulation in the rat aorta depending on Angiotensin II administration and vascular function	Angiotensin II infusion caused ferritin induction and iron deposition in the aortas Impairment of vascular function was mediated in part by enhancement of oxidative stress	[63]
	Relationship between chronic hemolysis/increased body iron burden and premature atherosclerosis	Carotid intima-media thickness in patients with thalassemia major was significantly increased compared with healthy controls	[64]

Diabetes and Obesity	Role of iron in adiposity using diabetic and obese mice model (KKAy) DFO treatment	DFO diminished fat iron and serum ferritin levels, reduced fat weight and adipocyte size, and reduced macrophage infiltration, superoxide production, NADPH oxidase activity, and mRNA of inflammatory cytokines	[65]
	Effect of intravenous iron preparation on the beta cells in isolated pancreatic islets	Exposure to iron resulted in a concentration-dependent oxidative stress and pancreatic islet cell death predominantly affecting beta cells	[66]

Renal fibrosis	Effect of an iron chelator (DFO) on renal fibrosis induced by unilateral ureteral obstruction in mice	DFO suppressed changes including macrophage infiltration, expression of collagen and inflammatory cytokines, activation of the TGF-beta1/Smad3 pathway, and tubulointerstitial fibrosis	[67]

Glaucoma	To investigate the association between dietary and total calcium and iron consumption with a diagnosis of glaucoma	Odds of glaucoma were increased in the population with higher total consumption of calcium and iron	[68]
	To study the effect of metal chelator EDTA on the rat optic nerve and retinal ganglion cells exposed to oxidative stress	EDTA ameliorated oxidative damage and inflammation, increased survival of retinal ganglion cell, and decreased demyelination of optic nerve	[69]

Systemic inflammatory response syndrome	To study the effect of DFO on acute hepatic ischemia induced SIRS in pigs	DFO completely blocked IL-6 production and lipid peroxidation and attenuated the development of SIRS and MOD	[70]
	To study the effect of DFO on acute hepatic ischemia induced SIRS in swine	DFO inhibited iron-catalyzed oxidative reactions, delayed the development of intracranial hypertension, and improved survival	[71]
	To test the hypothesis that inhibition of oxidative stress through iron chelation with DFO attenuates pulmonary injury caused by acute liver failure (ALF)	DFO reduced systemic and pulmonary oxidative stress during ALF, attenuated pneumonocyte necrosis, improved alveolocapillary membrane permeability, and prevented alveolar space collapse	[72]

Colitis	To evaluate the effect of selected iron chelators and antioxidants on protection of trinitrobenzene sulfonic acid (TNBS) induced colitis in rats	Maltol (iron chelator) was capable of protecting rat from TNBS induced colitis Kojic acid (iron chelator) and vitamin E (antioxidant) were not effective	[73]
